# Bridging the Gap in the Clinical Application of Advanced Parenteral Nutrition Formulations: From Efficacy to Real-World Challenges

**DOI:** 10.1016/j.advnut.2026.100621

**Published:** 2026-03-20

**Authors:** Mingdi Zhao, Xinlu Zhao, Ying Zhou, Jingyi Zhang, Lina Zhou, Shuang Zuo, Guifang Xu, Xiaojie Bian, Yun Zhu

**Affiliations:** 1 Department of Pharmacy, Nanjing Drum Tower Hospital, School of Basic Medicine and Clinical Pharmacy, China Pharmaceutical University, Nanjing, Jiangsu, China; 2 Department of Gastroenterology, Nanjing Drum Tower Hospital Clinical College of Nanjing Medical University, Nanjing, Jiangsu, China; 3 Department of Pharmacy, Nanjing Drum Tower Hospital, Nanjing Drum Tower Hospital Clinical College, Nanjing University of Chinese Medicine, Nanjing, Jiangsu, China; 4 Department of Pharmacy, Nanjing Drum Tower Hospital, Nanjing Drum Tower Clinical College of Xuzhou Medical University, Nanjing, Jiangsu, China; 5 Department of Pharmacy, Nanjing Drum Tower Hospital, Affiliated Hospital of Medical School, Nanjing University, Nanjing, Jiangsu, China; 6 Department of Gastroenterology, First Affiliated Hospital of Anhui Medical University, Hefei, Anhui, China

**Keywords:** parenteral nutrition, formulation, lipid emulsion, vitamins, stability, safety

## Abstract

Parenteral nutrition (PN) represents a critical therapeutic approach for patients unable to meet nutritional needs through oral or enteral routes. In recent years, the development of advanced PN formulations has accelerated, driven by improvements in lipid emulsion design, compound amino acid and micronutrient preparations, and multichamber bag (MCB) technologies. These innovations not only enhance safety, stability, and metabolic efficiency but also expand the capacity of PN to address increasingly complex clinical scenarios. Novel lipid emulsions derived from mixed-oil systems, structured triglycerides, and omega-3 polyunsaturated fatty acids provide both energy support and immunomodulatory effects. Advances in mixed micelles and nanoemulsion-based delivery systems have improved the solubility and chemical stability of labile vitamins and trace elements, whereas modern MCB systems reduce infection risk and simplify compounding procedures. Despite these advancements, significant challenges remain in translating laboratory progress into standardized clinical practice. Variability in formulation selection criteria, limited physicochemical stability in all-in-one systems, insufficient adaptability for pediatric and home parenteral nutrition populations, and inconsistencies in manufacturing quality and regulatory oversight all restrict broader clinical adoption. Moreover, the absence of unified evaluation standards for stability, compatibility, and clinical safety continues to hinder evidence-based optimization. This review summarizes the current progress and unresolved issues associated with advanced PN formulations, with particular focus on lipid emulsions, amino acids, vitamins, trace elements, and MCB technologies. Future directions include establishing multidimensional evaluation frameworks, promoting individualized and precision nutrition strategies, improving formulation processes, and strengthening global regulatory harmonization.

**Statement of significance**

This review advances the field by synthesizing emerging formulation technologies in parenteral nutrition with regulatory, stability, and patient-specific challenges that limit their routine use. Distinct from prior publications centered on single products or mechanisms, it highlights precision nutrition and standardized quality systems as critical, underexplored pathways for bridging innovation and clinical practice.

## Introduction

Parenteral nutrition (PN) is an essential modality of clinical nutritional support, indicated for patients who are unable to obtain adequate nutrients via oral or enteral routes for various reasons [1]. PN formulations consist of glucose, amino acids (AAs), lipid emulsions, trace elements, vitamins, electrolytes, and water, which are administered intravenously through a catheter directly into the bloodstream [1]. Since its introduction in the 1960s, the composition of PN formulations has undergone substantial evolution. Early PN regimens were primarily glucose based, with proteins supplied as large molecular forms that were poorly utilized, and the use of lipid emulsions was limited [2]. In addition, the supplementation of vitamins and trace elements lagged behind, failing to meet the patients’ nutritional needs [3,4].

With advances in clinical nutrition and pharmaceutical sciences, PN formulations have progressively evolved from basic nutritional support toward high-efficiency, precision-oriented, and safer advanced formulations. For example, novel lipid emulsions—such as medium- and long-chain triglyceride (LCT) emulsions, omega-3 (ω-3) fatty acid emulsions, and composite lipid emulsions—not only serve as energy sources but also play roles in delivering fat-soluble vitamins and modulating inflammation [5,6]. Moreover, innovative carriers such as mixed micelles have been introduced to improve the solubility and physicochemical stability of labile components, including vitamins [7,8]. In parallel, AA and trace element preparations have advanced toward standardized and compound formulations, simplifying clinical administration. Commercial multichamber bags (MCBs) reduce the risk of infection, shorten hospital stays, streamline prescription procedures, and effectively save medical resources and costs [9,10]. The development of these advanced formulations has therefore enhanced not only the diversity of PN compositions but also their efficacy, safety, and patient adherence [11,12].

**FIGURE 1:**
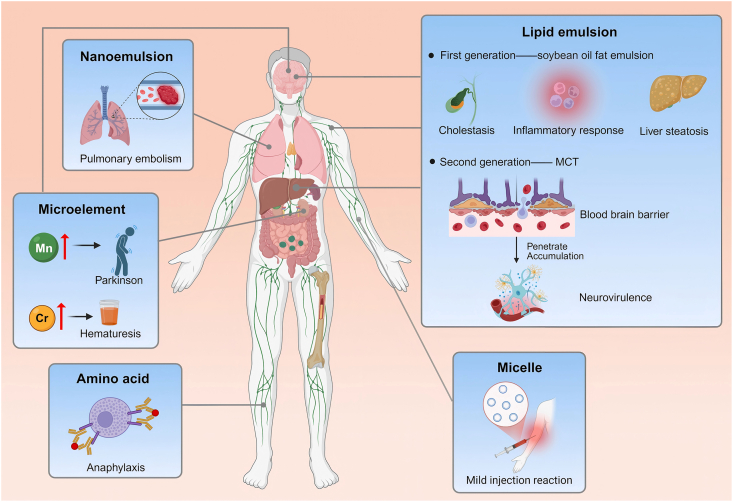
Complications associated with parenteral nutrition solutions. Figure created with BioRender.com. MCT, medium-chain triglycerides.

However, despite their promising potential in improving clinical outcomes and safety, the application of advanced PN formulations still faces a series of challenges (Figure 1). First, with the increasing diversity of PN formulations, discrepancies in component selection criteria, dosage forms, and safety profiles have become more pronounced [13]. For example, there is still no universal consensus on the optimal type of lipid emulsion; certain AA and vitamin preparations carry potential allergic risks; and the fixed composition and dosage of trace element formulations hinder precise supplementation. Second, issues related to physicochemical stability and infusion compatibility remain prominent, particularly in “all-in-one” (AIO) PN systems containing multiple components. In such systems, nanodispersed lipid emulsions may undergo particle size enlargement, aggregation, or phase separation, thereby increasing infusion risks [14]. Furthermore, PN patients represent a highly heterogeneous population with considerable interindividual variability in nutritional requirements, making standardized formulations insufficient for precision nutrition, particularly in pediatric and home parenteral nutrition (HPN) settings [15]. Lastly, the lack of unified quality evaluation standards and clinical safety monitoring systems continues to restrict the standardized implementation of these formulations [16]. Balancing clinical safety and efficacy with scalable production and rational use represents a critical direction for future research and policy development.

Therefore, this review focuses on the research progress and application challenges of advanced PN formulations, including lipid emulsions, mixed micelles, compound injections, and MCB technologies. It highlights current issues, bottlenecks, and future directions in formulation selection, optimization, stability enhancement, safety assurance, and adaptability for special patient populations, aiming to provide insights for technological innovation and the standardized application of advanced PN formulations.

## Core Components and Delivery Systems of PN

**FIGURE 2:**
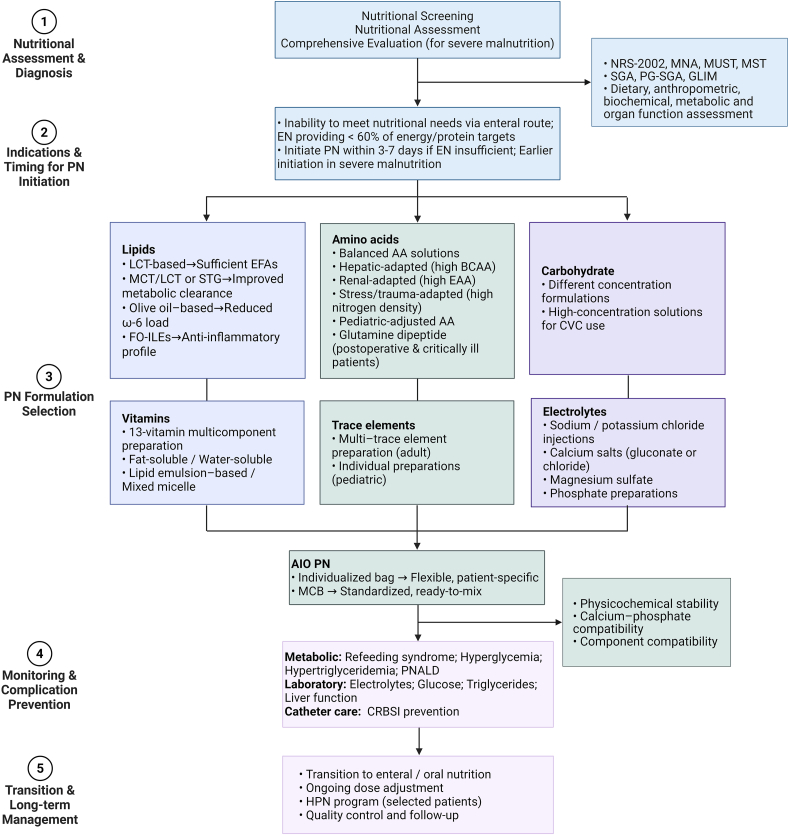
Clinical implementation roadmap of PN. AIO, all-in-one; AAs, amino acids; BCAA, branched-chain amino acids; CRBSI, catheter-related bloodstream infection; CVC, central venous catheter; EAAs, essential amino acids; EFA, essential fatty acid; EN, enteral nutrition; FO, fish oil; GLIM, Global Leadership Initiative on Malnutrition; HPN, home parenteral nutrition; ILE, intravenous lipid emulsions; LCT, long-chain triglyceride; MCB, multichamber bag; MCT, medium-chain triglyceride; MNA, Mini Nutritional Assessment; MST, Malnutrition Screening Tool; MUST, Malnutrition Universal Screening Tool; NRS, Nutritional Risk Screening; PG-SGA, Patient-Generated Subjective Global Assessment; PN, parenteral nutrition; PNALD, parenteral nutrition-associated liver disease; SGA, Subjective Global Assessment; STG, structured triglyceride.

To provide a structured overview of the core components and delivery systems of PN, we developed a clinical implementation roadmap (Figure 2). The framework integrates assessment, indication, formulation design, admixture strategy, monitoring, and transition planning into a continuous management pathway, emphasizing the interrelationship between nutrient composition, delivery modality, and safety surveillance.

### Lipid emulsions

Intravenous lipid emulsions (ILEs) are oil-in-water (O/W) nanoemulsions that do not contain any active pharmaceutical ingredients. Essentially, they are hydrophobic lipids stabilized into nanosized droplets within an aqueous phase by emulsifiers, typically phospholipids such as lecithin, allowing their safe intravenous administration [14]. As a core component of PN, ILEs provide not only a dense energy source and essential fatty acids (EFAs) but may also exert additional physiological effects by modulating inflammatory responses, immune function, and metabolic processes [17,18]. These effects have been associated with a lower risk of infection and sepsis, as well as shorter intensive care unit (ICU) and hospital length of stay [19,20]; in neonatal populations, they have further been linked to a reduced incidence of cholestasis and a shorter duration of respiratory support [21,22]. Since the introduction of the first LCT-based soybean oil emulsion (Intralipid) in 1961, the formulations of ILEs have continuously evolved. Successive generations have emerged, characterized by varying oil sources designed to optimize fatty acid profiles [5,23]. The first generation consists of pure LCT emulsions; the second generation includes medium-/LCT emulsions and structured triglyceride emulsions; the third generation comprises olive oil–based emulsions; and the fourth-generation features composite emulsions containing fish oil (FO) (Table 1). Notably, the recommendations for different types of ILEs vary across international guidelines. Although FO-containing ILEs, particularly SMOFlipid, are widely adopted in Europe and are extensively used in pediatric practice in both the United States and China, their positioning in adult clinical guidelines in these countries remains more cautious and heterogeneous [[48], [49], [50]]. The fatty acid composition and its biological characteristics remain the key determinants for ILE selection; however, no universally accepted optimal fatty acid profile has been established to date [24,51].

**TABLE 1:** Types and characteristics of major nutritional lipid emulsions.

Type	Advantages	Disadvantages	References
LCT-based emulsions	Rich in EFAs	High ω-6 PUFA content may exacerbate inflammation, immune dysregulation, and oxidative stress	[[24], [25], [26], [27]]
MCT/LCT emulsions	Mild effects; MCTs provide rapid energy, have lower proinflammatory potential, antioxidant and immunomodulatory effects, and minimal hepatic impact	—	[28,29]
Structured triglyceride emulsions	Rapid metabolism; improve hepatic function, nutrition, and immunity; more cost-effective than MCT/LCT	—	[[30], [31], [32], [33], [34]]
Fish oil–based emulsions	Rich in ω-3 PUFAs and α-tocopherol; anti-inflammatory, anticoagulant, antioxidant, and immunoregulatory effects; maintains EFA status at conventional doses in pediatric patients	Low content of ω-6 linoleic acid	[19,[35], [36], [37], [38]]
ω-3 fish oil MCT/LCT emulsions	Improved ω-6:ω-3 ratio; lower hepatic uptake; safe and well tolerated	—	[[39], [40], [41], [42]]
Olive oil/soybean oil emulsions	High in MUFAs; immunologically neutral metabolites; α-tocopherol in olive oil reduces lipid peroxidation	Biological advantages of olive oil are evident, but clinical benefits remain uncertain	[[43], [44], [45]]
Multioil emulsions	Proportional blending ensures EFA supply and exerts immunomodulatory, anti-inflammatory, antioxidative, and hepatoprotective effects	—	[46,47]

Abbreviations: EFA, essential fatty acid; LCT, long-chain triglyceride; MCT, medium-chain triglyceride; MUFA, monounsaturated fatty acid; PUFA, polyunsaturated fatty acid.

#### First-generation ILEs

LCT emulsions based on soybean oil, such as Intralipid and Nutrilipid, represent the earliest classic formulations used in PN. These emulsions are rich in ω-6 polyunsaturated fatty acids (PUFAs), with linoleic acid accounting for >50% of total fatty acids, resulting in an ω-6:ω-3 ratio of ∼7:1 [52]. Although soybean oil effectively supplies EFAs, its potential risks, particularly proinflammatory and immunomodulatory effects, including an increased risk of catheter-related bloodstream infections and PN-associated cholestasis, have attracted increasing attention [[53], [54], [55]]. After intravenous infusion, LCT droplets are hydrolyzed by lipoprotein lipase into free fatty acids, which are subsequently taken up by the liver and the reticuloendothelial system [52]. Because of the high ω-6 PUFA content, soybean oil emulsions promote the metabolism of arachidonic acid into prostaglandins, thromboxanes, and leukotrienes—mediators with proinflammatory effects—thereby intensifying inflammation and suppressing cell-mediated immunity [17]. Linoleic acid competitively inhibits the conversion of α-linolenic acid to eicosapentaenoic acid (EPA) and docosahexaenoic acid (DHA), reducing ω-3 fatty acid synthesis efficiency [17,56].

Due to their high degree of unsaturation and multiple double bonds, the PUFAs in soybean oil emulsions are inherently unstable [57]. Although lecithin forms a negatively charged interfacial layer that stabilizes lipid droplets, this electrostatic barrier can be compromised in the presence of multivalent cations (e.g., Ca^2+^ and Mg^2+^), increasing the risk of droplet aggregation and phase instability in AIO PN admixtures [[58], [59], [60]]. Furthermore, soybean oil emulsions contain relatively low concentrations of α-tocopherol, which limits their antioxidative capacity and increases susceptibility to lipid peroxidation on exposure to oxygen and light [61,62].

Clinically, lipid peroxides may activate macrophages and cause hepatocellular injury, whereas elevated concentrations of phytosterols can accumulate in the liver, contributing to cholestasis and an increased risk of intestinal failure-associated liver disease (IFALD) [[63], [64], [65]]. The European Society for Clinical Nutrition and Metabolism (ESPEN) guidelines recommend avoiding pure soybean oil–based emulsions in critically ill patients due to their possible proinflammatory effects [48,66]. Similarly, the German Society for Nutritional Medicine guideline recommends that PN in critically ill adult patients should include lipid emulsions with a reduced ω-6 fatty acid content [67]. Therefore, although first-generation soybean oil–based emulsions can meet short-term PN requirements for energy and EFAs, their high ω-6:ω-3 ratio, low antioxidant content, and elevated phytosterol concentrations restrict their suitability in patients with active inflammation, hypermetabolism, or prolonged PN dependence.

#### Second-generation ILEs

To overcome the unfavorable characteristics of soybean oil–based emulsions, second-generation ILEs were developed. The core strategy involves partially replacing soybean oil with alternative lipid sources—such as medium-chain triglycerides (MCTs)—that exert minimal effects on immune and inflammatory responses, thereby reducing ω-6 PUFA content and improving antioxidant properties. They have been reported to rapidly improve nitrogen balance, liver function, and overall nutritional status in critically ill or postoperative patients [31]. MCTs, derived from coconut oil or palm kernel oil, are rapidly metabolized in the liver and serve as an immediate energy source without increasing the synthesis of proinflammatory mediators, exerting little influence on immune function [68]. The short carbon chain and high degree of saturation confer enhanced oxidative stability and contribute to the formation of smaller and more uniform droplets [69]. In addition, MCTs can directly enter mitochondria for β-oxidation without requiring carnitine-mediated transport, providing rapid energy. Their plasma clearance half-life is significantly shorter than that of LCTs, thereby reducing the risk of lipid accumulation [29,70]. However, MCTs alone lack EFAs, and excessive metabolism may elevate serum ketone concentrations, limiting their use in patients with diabetes, ketosis, or metabolic acidosis [71]. Moreover, because MCTs are relatively water-soluble and can cross the blood–brain barrier, high concentrations may accumulate in the central nervous system and induce potential neurotoxicity [72].

On the basis of these properties, 2 major formulations were developed: MCT/LCT physical mixtures, typically composed of equal proportions of MCTs and soybean oil, and structured triglyceride emulsions (C6–24), synthesized through enzymatic or chemical re-esterification of equimolar amounts of MCTs and LCTs on a single glycerol backbone [73,74]. Both formulations exhibit a more compact molecular structure with greater steric hindrance, thereby reducing the risk of phase separation and improving droplet stability. A vitamin E–enriched MCT/LCT formulation (C8–24Ve, Lipofundin) has been developed to further enhance oxidative stability and reduce lipid peroxidation during infusion. In structured triglycerides, medium- and long-chain fatty acids are randomly distributed along the glycerol molecule, which effectively mitigates the neurotoxicity associated with free MCTs. These emulsions ensure an adequate supply of EFAs while decreasing lipid peroxidation and accelerating plasma clearance, making them more tolerable in patients under metabolic stress, infection, or hepatic dysfunction [75,76]. Compared with physical MCT/LCT mixtures, structured triglyceride emulsions demonstrate superior hepatic tolerance, improved nitrogen balance, and a smaller impact on plasma lipid profiles, thereby offering both metabolic and clinical advantages in catabolic or hepatically compromised patients [30,31,77].

#### Third-generation ILEs

Third-generation ILEs were designed to further minimize the proinflammatory potential and oxidative stress observed with earlier formulations. Olive oil–based emulsions, represented by ClinOleic, replace ∼80% of soybean oil with olive oil. Their primary component is ω-9 monounsaturated fatty acids (MUFAs), mainly oleic acid, which is naturally rich in the antioxidant α-tocopherol [78]. Oleic acid contains only 1 double bond, making it less susceptible to oxidation than linoleic acid. Once incorporated into cells, oleic acid is primarily integrated into membrane phospholipids, enhancing membrane fluidity and signal transduction, whereas its metabolism predominantly proceeds via β-oxidation with minimal production of inflammatory mediators [79]. Unlike ω-6 PUFAs, MUFAs are not readily converted into proinflammatory arachidonic acid derivatives and therefore exhibit a neutral inflammatory profile *in vivo* [80]. A meta-analysis of randomized trials showed that olive oil–based emulsions improve antioxidant and fatty acid profiles with safety comparable with soybean oil–based emulsions, whereas evidence for clear clinical outcome benefits remains limited [47]. Taken together, olive oil–based emulsions are considered to have relative advantages in terms of immune function, inflammation, coagulation, and oxidative stress, making them suitable for patients with impaired immunity or at high risk of immunosuppression [68].

#### Fourth-generation ILEs

To further optimize the ω-6 to ω-3 fatty acid ratio and explore ILEs with broader clinical benefits, FO-ILEs were developed [68]. FO is rich in ω-3 PUFAs, primarily EPA and DHA. Both EPA and DHA are not only essential structural components of cell membranes but also serve as precursors of specialized proresolving mediators (SPMs). These SPMs competitively inhibit arachidonic acid metabolism, thereby reducing the production of proinflammatory mediators such as prostaglandins and leukotrienes, while simultaneously promoting the biosynthesis of SPMs to facilitate inflammation resolution and maintain immune homeostasis [[81], [82], [83], [84]]. The rapid metabolic turnover of ω-3 PUFAs is associated with reduced serum triglyceride concentrations, improved lipid profile regulation, and favorable effects on endothelial function and hepatic metabolism [85]. Consequently, FO-ILEs are considered a physiologically favorable option [6,84,86,87].

Currently, 3 main formulations of FO-ILEs are available commercially. Omegaven, composed of 100% FO, provides an ω-3:ω-6 PUFA ratio of ∼8:1. Lipoplus, consisting of 50% MCT, 40% soybean oil, and 10% FO, provides a balance between rapid energy supply and anti-inflammatory effects. SMOFlipid is composed of 30% MCT, 30% soybean oil, 25% olive oil, and 15% FO. Its design concept aims to combine the advantages of multiple lipid sources: soybean oil ensures sufficient EFA provision; MCTs are rapidly metabolized for immediate energy; olive oil, rich in ω-9 MUFAs, is relatively immunologically neutral and less prone to lipid peroxidation; and FO contributes EPA and DHA, enhancing anti-inflammatory and immunomodulatory effects [74]. Both EPA and DHA contain 5–6 double bonds, rendering them highly flexible and oxidation-sensitive. α-tocopherol is added as an antioxidant to suppress free radical chain reactions and maintain droplet chemical stability [74]. In SMOFlipid, the combination of diverse oil sources forms a multimolecular interfacial structure that enhances steric hindrance and resistance to droplet aggregation. This formulation achieves a balance between energy provision, metabolic rate, antioxidative protection, and immunomodulatory capacity, demonstrating promising clinical utility in preterm infants, long-term PN recipients, and critically ill patients [88,89].

Multiple systematic reviews and meta-analyses have shown that FO-ILEs can reduce infection and sepsis incidence, shorten ICU and overall hospital stays, and decrease mechanical ventilation duration in critically ill or hospitalized patients [19,90,91]. Furthermore, pharmacoeconomic analyses conducted in China, the United States, and 5 European countries have indicated that FO-ILEs offer superior cost-effectiveness compared with traditional lipid emulsions [20,[92], [93], [94]]. Despite these advantages, FO-ILEs have not yet been prioritized in recent clinical guidelines, such as the American Society for Parenteral and Enteral Nutrition (ASPEN) 2022 recommendations. The guidelines acknowledge potential benefits for critically ill patients but emphasize that current studies exhibit substantial heterogeneity in design, inconsistent intervention regimens, short follow-up durations, and diverse outcome measures, which collectively limit the overall quality of evidence supporting FO-ILEs as a first-line lipid emulsion [25]. To facilitate clinical translation, these recommendations were synthesized into a scenario-based practical decision framework for lipid emulsion selection in PN (Table 2).

**TABLE 2:** Practical decision framework for lipid emulsion selection in PN.

Clinical scenario	Practical decision considerations	Guideline
Acute care, surgical and critically ill patients		
Postoperative patients requiring predominantly PN or combined EN/PN	PN including ω-3 fatty acids should be used when adequate EN is not feasible postoperatively (BM, HE) (grade B; strong consensus, 100% agreement)	ESPEN (2025) [95]
Adult surgical and critically ill patients	The use of ILEs containing fish oil should be considered during the first week of ICU admission, based on accumulated clinical evidence, safety, and cost-effectiveness	Expert Consensus (2025) [96]
Adult hospitalized patients requiring PN in the acute care setting	FO-containing ILE should be used as part of the lipid component of PN (high; strong; strong consensus)	Singapore (2025) [97]
Adult critically ill patients receiving PN	Either mixed-oil ILE or 100% soybean oil ILE may be used, and either FO-containing or non-FO-containing ILE may be used (low; weak)	ASPEN (2022) [25]
Critically ill adult patients requiring PN	PN should include lipid emulsions with reduced ω-6 fatty acid content (olive oil-based or mixed emulsions containing coconut and/or fish oils) (strong consensus 94%)	DGEM (2019) [67]
Long-term PN and chronic intestinal failure		
HPN or other long-term PN patients	FO-containing ILEs are preferred over pure soybean oil ILEs	Expert Consensus (2025) [98]
Adults with chronic intestinal failure	The choice of lipid emulsions should be individualized (grade B; strong consensus 97%); When higher lipid doses are required, mixed or alternative ILEs (olive oil, MCT, fish oil) should be considered (GPP; strong consensus 96%)	ESPEN (2023) [99]
PN-associated liver disease		
Adults with suspected PNALD	Lipid emulsions with a reduced ω-6/ω-3 fatty acid ratio can be used (grade 0; strong consensus 92%)	ESPEN (2019) [100]
Infants and children with PNAC	Lipid emulsions enriched with ω-3 fatty acids can be used (grade 0; strong consensus 100%)	ESPEN (2019) [100]
Patients with renal dysfunction		
Hospitalized patients with acute or chronic kidney disease	Routine use of ω-3 PUFA supplements or ω-3-enriched PN solutions is not supported due to insufficient evidence (GPP; strong consensus 95.8%)	ESPEN (2021) [101]
Pediatric and neonatal patients		
Preterm infants	No specific ILE composition is recommended (100% soybean oil vs. multicomponent ILE with or without FO) (very low; strong)	ASPEN (2023) [26]
Pediatric patients (including preterm infants and critically ill children)	For PN lasting more than a few days, pure soybean oil ILEs are not recommended, and composite ILEs with or without fish oil are preferred (LoE 1-, RG A, conditional recommendation for); In infants and children, 20% ILEs should be first choice (LoE 1-, RG B, strong recommendation for); Pure fish-oil ILEs are reserved for short-term rescue therapy in severe IFALD (LoE 3–4, GPP, conditional recommendation for)	ESPGHAN (2018) [102]

Abbreviations: ASPEN, American Society for Parenteral and Enteral Nutrition; BM, biomedical endpoint; DGEM, German Society for Nutritional Medicine; EN, enteral nutrition; ESPEN, European Society for Clinical Nutrition and Metabolism; ESPGHAN, European Society for Paediatric Gastroenterology Hepatology and Nutrition; FO, fish oil; GPP, good practice points; HE, health care economy endpoint; HPN, home parenteral nutrition; ICU, intensive care unit; IFALD, intestinal failure-associated liver disease; ILE, intravenous lipid emulsion; LoE, level of evidence; MCT, medium-chain triglyceride; PN, parenteral nutrition; PNAC, parenteral nutrition-associated cholestasis; PNALD, parenteral nutrition-associated liver disease; PUFA, polyunsaturated fatty acid; RG, recommendation grade.

Regionally, composite lipid emulsions containing multiple-oil sources have been widely adopted in Europe, whereas soybean oil–based emulsions remain predominant in the United States and China, reflecting differences in product availability and clinical acceptance [103,104]. Nonetheless, the traditional concept of using pure soybean oil emulsions has gradually been abandoned [105]. As a novel lipid emulsion combining energy provision with immunomodulatory effects, FO-ILEs have yet to receive universal guideline endorsement; however, their potential clinical value warrants continued attention. Future high-quality, multicenter randomized controlled trials employing standardized dosages and adequate intervention durations are needed to further elucidate their role in optimizing immune–metabolic homeostasis and improving clinical outcomes [106].

### Nanoemulsions

**FIGURE 3:**
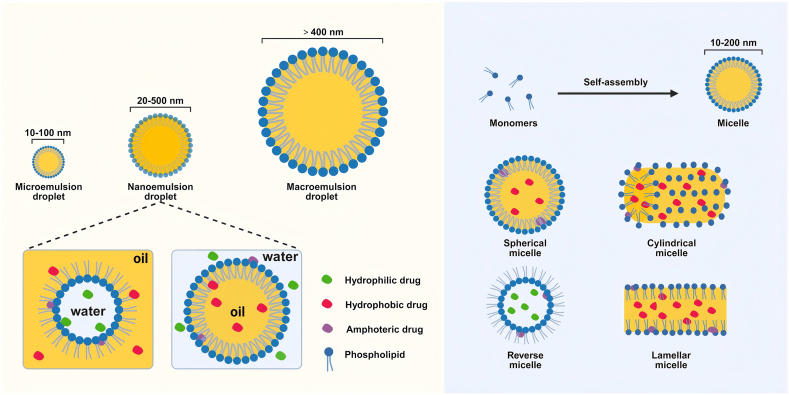
Spatial structure of nanoemulsions and micelles. Figure created with BioRender.com.

Nanoemulsions are colloidal dispersion systems composed of 2 immiscible liquids, typically characterized by droplet sizes ranging from 20 to 500 nm. They can be formulated as either O/W or water-in-oil (W/O) types (Figure 3) [107]. Due to their large specific surface area and high interfacial energy, nanoemulsions exhibit excellent solubilizing capacity, allowing simultaneous encapsulation of both hydrophilic and lipophilic active compounds while maintaining physicochemical stability at relatively low emulsifier concentrations [108]. Their unique physicochemical properties confer favorable dispersibility and biocompatibility *in vivo*, improving the solubility and bioavailability of lipophilic drugs. Moreover, their sustained and controlled release characteristics can reduce both dosing frequency and total administration dose. Nanoemulsions effectively protect encapsulated drugs from degradation induced by pH fluctuations, hydrolysis, and oxidation, and are suitable for various administration routes, including oral, intravenous, transdermal, and inhalation delivery [109]. As an efficient drug delivery system, nanoemulsions have demonstrated unique clinical value in drug transport, nutritional support, and targeted therapy, and are considered ideal carriers for achieving efficient, precise infusion and controlled drug release.

Nanoemulsions in PN serve not only as the primary source of energy and EFAs but also as carriers for the delivery of fat-soluble vitamins. Fat-soluble vitamins cannot be synthesized endogenously and must be obtained through external sources (except for vitamin D) [4]. Conventional vitamin preparations suffer from poor aqueous solubility and limited chemical stability; during infusion, they are prone to degradation under exposure to light, oxygen, and temperature fluctuations, leading to suboptimal dosing and formation of potentially harmful degradation products [110,111]. For example, vitamins A and E are highly sensitive to heat, light, humidity, and oxygen, making them vulnerable to oxidative degradation [112,113]. Similarly, vitamin D-3 is unstable in aqueous solution and easily degraded by temperature, pH, light, oxygen, and metal ions [114].

Commercial formulations such as Vitalipid utilize soybean oil as the oil phase and egg phospholipids as emulsifiers to encapsulate vitamins A, D, E, and K within lipid droplets, thereby meeting the basic requirements of PN patients [115]. By embedding vitamins in the oil phase and stabilizing them with phospholipid emulsifiers in the aqueous phase, nanoemulsions markedly improve vitamin solubility and stability, enhancing their controllability during compounding, storage, and infusion [116]. However, the value of nanoemulsions lies not only in their solubilization capability but also in their ability to achieve safe and predictable *in vivo* distribution and metabolism during long-term intravenous administration. Therefore, an ideal nanoemulsion for PN should ensure both physicochemical stability during preparation and storage, and efficient, controllable biodistribution and utilization *in vivo*.

**FIGURE 4:**
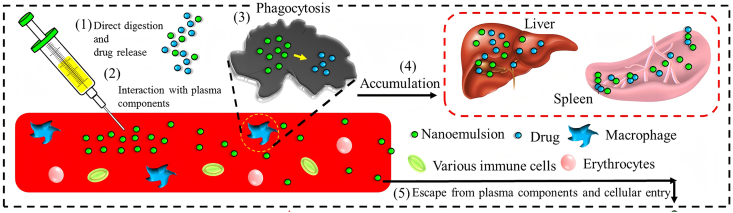
*In vivo* process of nanoemulsions after intravenous administration (adapted from [112]).

Nanoemulsions used in PN are thermodynamically unstable but kinetically stable O/W colloidal dispersions, typically with a mean droplet diameter (MDD) of 100–500 nm and a ζ-potential generally below −30 mV [107]. *In vivo*, nanoemulsions may release active components rapidly into the bloodstream or circulate as intact colloidal particles that interact with plasma proteins, erythrocytes, and immune cells (Figure 4) [112]. These critical quality attributes are closely associated with pharmacokinetic behavior: smaller and near-neutral droplets exhibit prolonged circulation and enhanced interstitial tissue penetration, whereas larger or highly charged particles are more readily cleared by the mononuclear phagocyte system and accumulate in the liver and spleen [117,118].

Experimental evidence consistently demonstrates size-dependent biodistribution of nanoemulsions. Fluorescence-tracking studies have shown that reducing droplet size prolongs systemic circulation, with distinct organ distribution patterns depending on particle diameter [119]. Similarly, nanoemulsion-based delivery of vitamin K-1 significantly alters its tissue distribution, resulting in increased tissue exposure in major organs compared with conventional injections [120]. Collectively, these findings support the role of nanoemulsion formulation in modulating *in vivo* exposure and tissue distribution of lipophilic vitamins.

Nanoemulsions exhibit excellent biocompatibility, low systemic toxicity, and well-established manufacturing techniques. Their advantages lie in significantly improving the solubility and stability of labile components, reducing adsorption to infusion tubing, photodegradation, and oxidative decomposition, as well as minimizing adverse reactions associated with traditional solubilizers such as propylene glycol, ethanol, or surfactants [107,121]. Several formulation studies have demonstrated that vitamin-loaded nanoemulsions with optimized particle size, ζ-potential, and encapsulation efficiency exhibit favorable *in vitro* stability and *in vivo* safety profiles [122,123]. However, nanoemulsions also have inherent limitations. Their thermodynamic instability may lead to the formation of large oil droplets [percentage of fat residing in globules >5 μm (PFAT5) >0.05%] during storage or infusion, thereby increasing the risk of fat embolism [112,124]. Furthermore, rapid clearance by the reticuloendothelial system may shorten effective exposure time and limit delivery efficiency [125]. In addition, the compatibility between excipients and coloaded drugs is a crucial variable in formulation design. Interfacial competition can alter droplet size and ζ potential, subsequently affecting biodistribution and toxicity profiles.

Researchers often use commercial nanoemulsions as the basis for exploring drug delivery applications, and these studies have demonstrated the feasibility and safety of nanoemulsions as drug delivery systems. Commercial PN lipid emulsions, such as SMOFlipid, Intralipid, ClinOleic, and Lipofundin, have been extensively validated in clinical settings for safety, injectability, and long-term stability. Therefore, they are frequently employed as lipid carrier models for formulation optimization and functional enhancement. For instance, honokiol-loaded nanoemulsions based on SMOFlipid or Lipofundin achieved high encapsulation efficiency and stable particle characteristics during refrigerated storage, while maintaining injectability and acceptable hemocompatibility, suggesting potential benefits in mitigating PN-associated liver injury [126,127]. Similarly, paclitaxel incorporation into Intralipid or ClinOleic significantly enhanced aqueous solubility and *in vivo* stability, with the ClinOleic-based formulation showing improved antitumor efficacy and selectivity toward glioblastoma cells [128]. In addition, Intralipid-based multidrug nanoemulsions enabled effective tumor growth inhibition and immune modulation in murine melanoma models without apparent systemic toxicity [129]. Antibiotic-loaded systems, such as ciprofloxacin nanoemulsions formulated with ClinOleic or Intralipid, also exhibited long-term physicochemical stability under refrigerated conditions [130]. These findings suggest that the formulation development of commercially available nanoemulsions as delivery vehicles provides a translational basis in terms of solubilization, stability, and preliminary safety.

### Mixed micelles

Mixed micelles are nanoscale self-assembled delivery systems composed of bile salts and phospholipids as surfactants (Figure 3), and have been widely applied in recent years for the delivery of poorly soluble drugs [131]. In the field of PN, mixed micelles are employed in both multivitamin and single-vitamin injectable formulations—for example, Cernevit, which contains 12 vitamins, and the vitamin K-1 formulation Konakion MM. These systems enable stable vitamin delivery without relying on polysorbate 80 (Tween-80) or polyoxyethylene castor oil (Cremophor EL), and are particularly suitable for patients in whom lipid emulsions are contraindicated [132,133]. Cernevit forms a true mixed micellar system based on glycocholic acid and lecithin, allowing both water- and fat-soluble vitamins to coexist in a single vial and eliminating the need for dual-vial packaging. The micellar system remains stable in PN admixtures with or without lipid emulsions and can be reconstituted with 5 mL of solvent for intravenous injection or 2.5 mL for intramuscular injection [134].

By contrast, Soluvit and Infuvite do not contain mixed micelles but adopt different solubilization strategies. Soluvit contains ethylenediaminetetraacetic acid (EDTA), which can chelate metal ions, inhibit oxidation, and prevent salt precipitation, thereby maintaining the activity, clarity, and safety of water-soluble vitamins during infusion [135]. Infuvite employs polysorbate 80 as an emulsifier for lipid-soluble vitamins, enabling the combined delivery of water- and fat-soluble components, but polysorbates are susceptible to degradation and have been associated with particle formation and hypersensitivity or immunogenicity risks [136,137]. In clinical use, strict control of electrolyte concentrations and storage conditions (2–8°C, protected from light), as well as allergy screening, is necessary to minimize adverse events.

The typical structure of a mixed micelle consists of a hydrophobic core surrounded by a hydrophilic shell, allowing efficient encapsulation of lipophilic drugs while maintaining stable dispersion in aqueous media, thereby markedly enhancing solubility and bioavailability [138]. Compared with traditional solubilizers composed of a single surfactant, mixed micelles exhibit lower critical micelle concentration (CMC) values and stronger solubilizing capacity [139,140]. Because both bile salts and phospholipids are endogenous to the human body, mixed micelles generally demonstrate excellent biocompatibility and safety, making them an ideal platform for the delivery of various poorly soluble drugs, such as lenvatinib, silybin, and curcumin [[141], [142], [143]].

In contrast, certain vitamin injections employ Tween-80 or Cremophor EL as solubilizers, for which hypersensitivity reactions are frequently reported, typically presenting as non–IgE-mediated anaphylactoid responses [[144], [145], [146], [147]]. Due to their relatively high CMC values, these low-molecular-weight surfactants must be used at high concentrations to maintain drug solubility, which not only increases the risk of allergic and toxic reactions but may also lead to drug precipitation when the concentration falls below the CMC [148]. Postmarketing surveillance and experimental studies consistently indicate that adverse reactions are largely attributable to the excipients rather than the vitamins themselves, and that removal of Tween-80 or Cremophor EL substantially reduces their incidence [[149], [150], [151]]. Randomized clinical trials further demonstrate that mixed micelle formulations provide more stable pharmacokinetic profiles and improved tolerability, with no evidence of hemolysis or hepatic dysfunction during intravenous administration [[152], [153], [154]].

The advantages of mixed micelles extend beyond the reduction of infusion-related reactions and avoidance of organic solvent–associated risks; they also derive from their unique thermodynamic and kinetic stability [155]. The hydrophobic core of the mixed micelles can effectively protect encapsulated lipophilic vitamins from the surrounding aqueous environment, thereby prolonging circulation time and improving pharmacokinetic performance [156]. These characteristics have led to their increasing clinical recognition as an optimal platform for the delivery of fat-soluble vitamins.

However, current mixed micelle-based multivitamin formulations do not include vitamin K. For PN patients receiving warfarin therapy, vitamin K–free preparations are often preferred to allow flexible anticoagulation management. According to the ASPEN guidelines, PN patients treated with warfarin may receive multivitamin products with or without vitamin K to facilitate control of anticoagulation when needed [157], whereas the Australasian Society for Parenteral and Enteral Nutrition guideline also notes that patients receiving lipid-based PN usually do not require extra vitamin K supplementation [158]. Therefore, 12-vitamin formulations without vitamin K remain appropriate for PN patients under anticoagulation therapy. Nevertheless, such formulations still fail to fully meet the 13-vitamin supplementation scheme recommended by clinical guidelines [4,[158], [159], [160]]. Future research should focus on developing mixed micelles composite formulations containing vitamin K, systematically evaluating the effects of bile salt/phospholipid ratios on physicochemical stability and PN compatibility, and establishing long-term safety monitoring and clinical efficacy assessment frameworks to facilitate the standardized application of mixed micelle-based formulations in vitamin injections and PN support.

**SCHEME:**
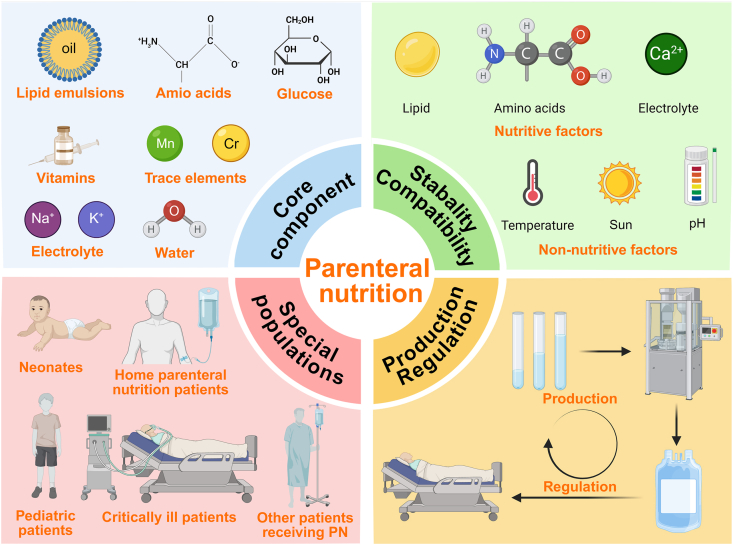
The characteristics, production process, and application scenarios of high-end parenteral nutrition preparations. PN, parenteral nutrition.

### Compound formulations

#### Amino acids

AAs are the fundamental building blocks of proteins and play critical roles in maintaining nitrogen balance, promoting tissue repair, regulating immune function, and synthesizing hormones and enzymes [161]. Compound AA injections constitute the primary nitrogen source in PN, supporting positive nitrogen balance and muscle protein synthesis. Commercial products are mainly categorized into balanced AA injections (e.g., 18AA, 18AA-Ⅱ) and disease-specific formulations (e.g., 20AA for liver disease, 9AA for renal disease, 18AA-Ⅶ for trauma), which adjust the ratio of branched-chain to aromatic AAs or limit specific AAs to meet distinct metabolic demands [162].

Aqueous injection solutions remain the standard formulation for PN AAs, dictated by both their physicochemical properties and clinical requirements. AAs, as zwitterionic electrolytes, exhibit high water solubility, low-molecular weight, and intrinsic stability; simple pH and osmolarity adjustments suffice to produce isotonic solutions compliant with pharmacopoeial standards. Clinically, the primary requirement is rapid and uniform distribution as a nitrogen source to support protein synthesis, which does not necessitate carrier-mediated delivery or sustained-release systems. From the perspectives of necessity, safety, and cost-effectiveness, introducing complex carrier systems provides no substantial clinical benefit and may increase production costs and safety risks, such as immunogenicity or toxic accumulation. Therefore, conventional AA injections remain the optimal choice for meeting baseline nitrogen requirements and are fully compatible with clinical practice.

Nevertheless, safety concerns persist for current compound AA injections. To prevent oxidative degradation, most formulations include sodium metabisulfite or sodium bisulfite as antioxidants, which carry potential hypersensitivity risks [163]. A systematic review of Chinese literature (1985–2010) reported 71 cases of anaphylactic shock and 7 deaths related to compound AA injections, with pregnant women, the elderly, and patients with asthma identified as high-risk populations [164]. Hypersensitivity reactions have also been reported in neonates exposed to bisulfite-containing formulations, which resolved after switching to sulfite-free alternatives [165]. Comparative evaluations of balanced AA injections have shown substantial differences among products in sulfite content, nutritional composition, and cost, leading to recommendations favoring formulations with lower sulfite concentrations and more appropriate essential AA ratios [166]. Recently, several sulfite-free formulations (e.g., 18AA-V-SF, 14AA-SF, and dipeptide-containing products) have been developed, reflecting a trend toward formulation optimization to improve safety and minimize hypersensitivity reactions. However, despite their potential advantages, the relatively high cost and the limited evidence regarding their clinical nutritional efficacy and interaction with AA compositions warrant further investigation.

Despite the variety of AA formulations, their compositions still fall short of fully supporting individualized clinical nutrition. Commercial compound AA injections exhibit significant differences in the proportions of essential and branched-chain AAs, as well as specific AA doses (e.g., glutamine, arginine, cysteine), lacking a standardized framework. Most ratios remain based on nitrogen balance studies from the previous century, without fully accounting for AA metabolism dynamics under different disease states [167]. The WHO, United Nations University (UNU), and FAO have jointly established recommended intakes and proportions of essential AAs for humans, providing a theoretical reference for formulation design. However, evidence suggests that meeting total essential AA requirements alone may be insufficient to optimize functional outcomes, such as muscle protein synthesis [168,169]. They suggested aligning the EAA profile closer to that of whey protein to enhance muscle anabolic efficiency. However, this recommendation lacks validation from large-scale randomized controlled trials, and the direct impact of AA ratios on clinical outcomes remains insufficiently evidenced.

Table 3 presents the AA composition of 5 commonly used parenteral AA formulations. Compared with the WHO/FAO/UNU recommended daily requirements for EAAs in adults, Aminoplasmal, Novamin, and Aminoven contain EAA concentrations ∼2–4 times higher than the reference values. Among them, Aminoplasmal shows the most comprehensive advantages—it is free of sodium metabisulfite, has the highest AA concentration, and can rapidly correct negative nitrogen balance during hypercatabolic states without increasing fluid load. 14AA-SF, although also free of sulfites, lacks 6 AAs, including phenylalanine, tyrosine, and glutamic acid, resulting in an incomplete AA profile; thus, it is suitable only for special situations requiring restriction of aromatic AAs. 18AA-V-SF is likewise free of sulfites; however, it has the lowest total nitrogen content and requires a 2–3-fold larger infusion volume to provide an equivalent nitrogen supply, which may impose excessive fluid load.

**TABLE 3:** Essential and total nonessential amino acid content in commonly used parenteral amino acid formulations in adults.

Amino acid	Product (g/500 mL)					Essential amino acid requirements^1^ (g/d)
	10% Aminoplasmal	8.5% Novamin	11.4% Aminoven	3.2% 18AA-V-SF	8.5% 14AA-SF	
Isoleucine	4.4	2.1	4.55	0.85	2.95	1.2
Leucine	6.8	2.95	6.45	1.895	3.85	2.34
Lysine	5.3	4.75	5	1.665	4.35	1.8
Methionine	0.6	2.1	2.2	0.53	2.25	0.9
Cysteine	0.3	0.1	0.175	0.22	0	
Phenylalanine	0.8	2.95	3.5	1.415	2.4	1.5
Tyrosine	0.35	0.1	0.2	0.055	0	
Threonine	2.3	2.1	3.75	0.985	1.7	0.9
Tryptophan	0.75	0.7	0.65	0.195	0.65	0.24
Valine	5.3	2.75	7	0.68	2.8	1.56
Arginine	4.4	4.2	4.5	1.445	4.05	—
Histidine	2.35	2.5	2.5	1.23	1.2	0.6
Glycine	3.15	2.95	3.5	1.62	5.95	—
Alanine	4.15	6.1	3.55	0.94	3	—
Proline	3.55	2.5	2.5	0.5	4.75	—
Asparatic acid	1.25	1.25	0.5	0.575	0	—
Asparagine	0.275	0	0	0	0	—
Glutamic acid	2.85	2.1	0.25	0.985	0	—
Ornithine	0.83	0	0	0	0	—
Serine	1.85	1.7	0.85	0.335	2.5	—
Nitrogen content (g/500 mL)	7.65	7	7.5	2.58	6.78	—
Osmotic pressure (mOsm/L)	875	850	—	320	850	—

Abbreviation: UNU, United Nations University.

1 Daily essential amino acid requirements were calculated for adults weighing 60 kg, according to the reference values recommended by WHO/FAO/UNU [170].

Table 4 summarizes the AA composition of commonly used pediatric parenteral AA formulations. In contrast to adult formulations, pediatric AA solutions are designed to meet the increased requirements for growth and development and therefore contain a higher proportion of essential and conditionally EAAs. Formulations such as Trophamine, Aminosyn II, and Travasol are relatively enriched in branched-chain AAs, arginine, and histidine, and some include taurine to support neonatal and pediatric metabolic needs. Nevertheless, the composition of parenteral AA formulations still has room for optimization, with future efforts focusing on formulations better tailored to metabolic requirements.

**TABLE 4:** Essential and total nonessential amino acid content in commonly used parenteral amino acid formulations in pediatrics.

Amino acid	Product (g/500 mL)			Essential amino acid requirements (mg/kg/d) [170]				
	10% Trophamine	8.5% Aminosyn II	10% Travasol	0.5 (y)	1–2 (y)	3–10 (y)	11–14 (y)	15–18 (y)
Isoleucine	4.1	2.805	3	36	27	23	22	21
Leucine	7	4.25	3.65	73	54	44	44	42
Lysine	4.1	4.465	2.9	64	45	35	35	33
Methionine	1.7	0.73	2	31	22	18	17	16
Cysteine	<0.08	0	0					
Phenylalanine	2.4	1.265	2.8	59	40	30	30	28
Tyrosine	1.2	1.15	0.2					
Threonine	2.1	1.7	2.1	34	23	18	18	17
Tryptophan	1	0.85	0.9	9.5	6.4	4.8	4.8	4.5
Valine	3.9	2.125	2.9	49	36	29	29	28
Arginine	6	4.325	5.75	—	—	—	—	—
Histidine	2.4	1.275	2.4	22	15	12	12	11
Glycine	1.8	2.125	5.15	—	—	—	—	—
Alanine	2.7	4.22	10.35	—	—	—	—	—
Proline	3.4	3.07	3.4	—	—	—	—	—
Asparatic acid	1.6	2.975	0	—	—	—	—	—
Asparagine	0	0	0	—	—	—	—	—
Glutamic acid	2.5	3.135	0	—	—	—	—	—
Ornithine	0	0	0	—	—	—	—	—
Serine	1.9	2.25	2.5	—	—	—	—	—
Taurine	0.125	0	0	—	—	—	—	—
Nitrogen content (g/500 mL)	7.75	3.25	8.25	—	—	—	—	—
Osmotic pressure (mOsm/L)	866	896	998	—	—	—	—	—

Glutamine is considered a conditionally EAA whose demand under severe stress (e.g., trauma, infection, major surgery) exceeds endogenous synthesis capacity. Supplementation can improve nitrogen balance, maintain intestinal barrier function, and modulate immune responses [171]. Specifically, glutamine enhances monocyte surface human leukocyte antigen-DR (HLA-DR) expression, increases neutrophil phagocytic capacity, and promotes heat-shock protein synthesis, thereby strengthening defense against inflammation and oxidative stress [172]. However, free glutamine exhibits poor stability in aqueous solutions, decomposing into toxic ammonia and pyroglutamate, with limited solubility, restricting its direct addition to PN [173]. To overcome these limitations, synthetic dipeptides such as L-alanyl-L-glutamine have been developed, offering high solubility, thermal stability, and rapid enzymatic hydrolysis *in vivo* to release bioactive glutamine efficiently and safely [174]. The N-terminal conjugation protects the α-amino group, allowing heat sterilization and safe incorporation into other AA injections or MCB [175]. Multiple studies have demonstrated that supplementation with these dipeptides effectively maintains muscle glutamine stores, improves nitrogen balance, enhances intestinal mucosal integrity, and reduces infection incidence [176,177]. This represents a substantial advance in the functionalization and precision delivery of AA formulations, making dipeptide-based glutamine the closest current example of advanced AA formulations.

#### Trace elements

Trace elements are essential micronutrients present at <0.01% of total body mass, including copper, zinc, iron, chromium, cobalt, manganese, fluoride, molybdenum, selenium, and iodine [4]. These elements play pivotal roles in maintaining metabolic homeostasis, immune function, and antioxidant defense. Both deficiency and excess intake can lead to a range of clinical issues, such as impaired growth and development, reduced immunity, organ dysfunction, and increased risk of chronic diseases [178,179]. Given the Food and Drug Administration (FDA) resources and the long-standing global shortage of trace element formulations, many commercial products remain the sole source of micronutrient supplementation for PN patients [180]. These composite formulations provide basal doses in a single preparation, simplifying compounding, reducing dosing errors, and minimizing the risk of microbial contamination [3]. Similar to AA solutions, trace elements possess well-defined physicochemical properties and good water solubility, making conventional aqueous injection the most direct and cost-effective approach for daily supplementation. Current formulation development focuses on optimizing existing products to enhance safety and applicability.

Current formulations deliver multiple trace elements at fixed doses, which may not be appropriate for all patients, particularly in pediatrics. In clinical practice, pediatric PN increasingly favors the use of single-trace element preparations, most commonly copper, zinc, and selenium, to allow more precise adjustment according to age, weight, organ function, and disease state [181]. Certain clinical scenarios require adjusting the reliance of specific elements on composite formulations [182]. ASPEN highlights that commercially available trace element products may provide excessive amounts of manganese, copper, and chromium, whose potential toxicity warrants attention [183]. Accordingly, several professional societies have issued recommendations for daily trace element intake in adult and pediatric PN patients [4,159,160,181,184]. For example, patients with cholestasis require reduced manganese supplementation [185]; severely burned patients need increased copper and decreased selenium intake [186]; patients with significant gastrointestinal losses require higher zinc and selenium supplementation [187]. For short-term PN (<4 wk) and clinically stable patients, weight-based dosing is generally appropriate. In pediatric and HPN populations, supplementation should be individualized, taking into account patient weight, clinical condition, and guideline recommendations [182,188]. Although fixed-dose composite formulations facilitate clinical use, their lack of flexibility limits application in precision nutrition and exposes a disconnect between current formulation design and personalized medicine principles.

On the other hand, nonintentional contamination of trace elements in PN products is a critical concern. Contamination levels are influenced by raw material sources, production batches, manufacturing processes, storage conditions, and compounding practices [189]. Excess trace element contamination may lead to toxic exposure, particularly for aluminum, manganese, and chromium [[190], [191], [192]]. Manganese and chromium are now widely recognized as contaminants of PN solutions, originating from raw materials and manufacturing processes rather than from intentional supplementation [192]. Although the FDA has imposed mandatory limits on aluminum content in PN products and requires labeling, a study in children receiving HPN reported generally elevated serum and urinary aluminum concentrations, with only 12% of patients exhibiting serum aluminum <5 μg/L [193]. Manganese contamination may lead to hypermanganesemia with basal ganglia deposition and Parkinson-like neurotoxicity, prompting recommendations against routine manganese supplementation in neonates and HPN patients [[194], [195], [196], [197], [198]]. Excessive chromium intake may impair renal function, necessitating a reduction in recommended PN chromium doses [199]. A Canadian pediatric study found that chromium and manganese concentrations in PN formulations substantially exceeded recommended concentrations [192]. In summary, optimization of current trace element formulations focuses on 2 key aspects: *1*) reconciling fixed-dose formulations with individualized clinical needs, and *2*) minimizing contamination risk through improved manufacturing processes and quality control standards.

### All-in-one parenteral nutrition

With the standardization of PN, AIO PN formulations have gradually replaced the traditional multibottle infusion approach. The core concept of AIO PN is to premix AAs, glucose, lipid emulsions, electrolytes, and micronutrients in a single bag at predetermined ratios, providing comprehensive nutritional support via intravenous administration [200]. Compared with conventional single-bottle infusions, AIO PN not only supports anabolic metabolism but also simplifies compounding procedures and reduces the risk of microbial contamination and bloodstream infections [9,201]. Depending on patient needs and available healthcare resources, AIO PN can be provided as individualized composite bags or MCB [201]. Individualized composite bags are prepared on demand by hospital pharmacies or community compounding facilities, allowing precise tailoring to patient-specific nutritional requirements, but are associated with higher compounding costs, limited stability, and greater technical demands [2]. In contrast, MCBs employ innovative compartmentalized designs, maintaining component stability while allowing mixing immediately before administration. Although their standardized formulations offer less flexibility, MCBs help address resource limitations in certain healthcare settings [16,202,203].

Clinical studies indicate that MCBs demonstrate comparable efficacy and safety to individualized composite bags in adult patients, while potentially offering greater cost-effectiveness [204]. Nevertheless, the broader implementation of MCBs requires rigorous oversight of product quality, storage stability, and infusion safety [9,205]. Commercially available MCBs include dual-chamber and triple-chamber bags [201]. Dual-chamber bags typically contain glucose and AAs, whereas triple-chamber bags additionally include lipid emulsions. Despite improvements in convenience and safety, these products are supplied without premixed vitamins and trace elements, necessitating additional supplementation before clinical use, which increases the management burden for micronutrients [9]. To address this limitation, Fukatsu et al. [206,207] developed the first peripheral AIO PN formulation containing multiple vitamins (OPF-105). Phase I–III clinical trials demonstrated that OPF-105 effectively improved lipid metabolism, maintained plasma vitamin concentrations, and exhibited a favorable safety profile [206,207]. These findings suggest that stable premixing of micronutrients in MCBs could enhance the overall adaptability and safety of AIO PN.

In pediatrics, particularly in preterm and critically ill infants, the routine use of lipid-containing AIO PN admixtures remains limited. Owing to the high calcium and phosphate requirements in this population and the narrow solubility window of calcium–phosphate salts, precipitation may occur without being visually detectable in AIO PN, which compromises real-time safety assessment [208]. Due to the lack of robust data in neonates and the multiple unique risks in this age group, 3-in-1 PN solutions are generally avoided [209]. Nevertheless, several studies have begun developing pediatric AIO PN formulations suitable for different age groups and body weights [[210], [211], [212]]. Evidence indicates that optimizing PN composition and storage conditions can enhance clinical utility. Increasing the industrial utilization of triple-chamber bags has significantly improved clinical outcomes in preterm infants, optimized resource use, and reduced hospital costs, demonstrating favorable cost-effectiveness [213]. Therefore, in settings with limited pharmacy compounding capabilities, promoting pediatric-specific MCBs can further optimize PN practice [203]. Overall, MCBs have made notable progress in enhancing PN safety, streamlining workflow, and improving resource efficiency. However, challenges remain regarding the balance between standardized formulations and individualized nutrition, intercomponent compatibility, and postcompounding stability, which warrant further research and refinement.

## Real-World Challenges

Despite advances in PN formulations, their effectiveness in real-world clinical practice is limited by multiple barriers. A summary of these barriers, their impact on effectiveness, and proposed solutions is presented in Table 5.

**TABLE 5:** Real-world barriers limiting the effectiveness of PN.

Barrier category	Key barriers	Impact on effectiveness	Proposed solutions
Formulation-related	Physicochemical instability and incompatibility in AIO admixtures; lack of consensus on component selection; fixed-dose PN formulations	Degradation of labile nutrients; imprecise nutrient delivery	Stability and compatibility testing; wider adoption of MCBs; flexible formulation design
Manufacturing and supply	Shortages of key components; batch-to-batch quality variability; limited availability of specialized formulations	Treatment interruption; inconsistent clinical outcomes; restricted access for high-need populations	Strengthened quality control and regulatory oversight; alternative sourcing strategies; incentives for specialized formulations
Healthcare system	Limited implementation of nutrition support teams; prescribing and compounding complexity; nonstandardized monitoring	Inappropriate PN prescriptions; higher complication rates; failure to achieve nutritional targets	Multidisciplinary NST management; electronic prescribing with decision support; standardized compounding and monitoring protocols
Patient and caregiver factors	Inadequate training for HPN; challenges in aseptic handling; psychosocial and financial burden	Increased catheter-related infections; poor adherence; reduced quality of life	Structured HPN education programs; long-term follow-up with remote support; integrated psychosocial and financial counseling
Economic and policy	High cost of advanced formulations; restrictive reimbursement policies	Inequitable access; low adoption of innovative products; delayed market diffusion	Cost-effectiveness and budget-impact analyses; evidence-based reimbursement updates
Evidence and guidelines	Limited long-term outcome data; heterogeneous study designs; delayed guideline updates	Clinical uncertainty; practice variability; slow translation of innovation	Pragmatic multicenter RCTs; standardized research methodologies; dynamic guideline updates based on living evidence

Abbreviations: AIO, all-in-one; HPN, home parenteral nutrition; MCB, multichamber bag; NST, nutrition support team; PN, parenteral nutrition; RCT, randomized controlled trial.

### Stability and compatibility

Stability encompasses physical, chemical, and microbiological stability, whereas compatibility refers to the interactions among different components, including active ingredients, excipients, container materials, and concomitant medications [8]. PN admixtures are among the most complex pharmaceutical preparations used in clinical practice, and their stability and compatibility are critical determinants of therapeutic efficacy and patient safety [214]. Instability or incompatibility may result in serious complications such as catheter occlusion, venous thrombosis, and local or systemic inflammatory reactions [215].

The stability and compatibility of PN solutions are influenced by multiple factors, which have been summarized in several reviews (Table 6) [8,214,216]. Current studies have investigated the effects of both formulation-related factors, such as the type and concentration of lipid emulsions, electrolytes, and AAs, and nonpharmaceutical factors, including temperature, light exposure, and packaging materials. In addition, compatibility issues between PN admixtures and commonly coadministered drugs (e.g., antibiotics, insulin, high-dose vitamin C), as well as administration via Y-site connections, have also been explored. These investigations not only deepen our understanding of the mechanisms underlying PN stability and compatibility but also provide theoretical and practical guidance for clinical PN therapy. However, with the increasing use of advanced PN formulations, more practical challenges have emerged alongside the growing complexity of PN compositions and the heterogeneity of research methodologies. These aspects are discussed in the following sections.

**TABLE 6:** Major factors influencing the stability and compatibility of parenteral nutrition admixtures (·adapted from [8]).

Category		Factor	Effect on PNS stability
Factors affect PNS stability	Nutritive factors	Amino acids	• Buffering effect: higher concentrations improve emulsion stability and reduce Ca–P precipitation. • Some acidic AAs cause creaming and cracking of the emulsion. • Oxidizing agents like cysteine affect the bioavailability of easily oxidized vitamins such as ascorbic acid.
		Glucose	• Concentrated glucose solutions cause disruption of the lipid phase. • Maillard process may affect the bioavailability of AAs and may even produce toxic compounds. • Lower glucose concentration potentially increases Ca–P precipitation.
		Lipid	• Emulsifying agents, such as lecithin phospholipid, help maintaining stability of LEs. • LEs in PNS are most stable at a pH of 8. • Composite lipid formulas are more stable compared with purely long-chain triglycerides formula.
		Electrolytes	• Overall concentration, individual cation burden, and electrolytes ratios have important effects on the stability of the solution. • High concentration of cations destabilizes lipid droplets. • Ca–P precipitation is influenced by multiple factors. Low pH and low calcium and phosphate concentrations prevent precipitation and maintain solubility.
		Vitamins	• The least stable of all nutrients in PNS. Stability could be affected by photodegradation, oxidation, and interaction with storage material. • Minimizing contact time, by adding vitamins to PNS immediately before use, may help to reduce stability and compatibility effects.
		Trace elements	• Some of trace elements may form insoluble salts. • Copper can exacerbate degradation of vitamins. • Positively charged trace elements may have a role in neutralizing the negative charge of the lipid emulsion and cause instability.
	Nonnutritive factors	pH	• Acidic pH values may jeopardize the integrity and stability of LEs, whereas alkaline pH ideally favors the stability of the emulsion and maintains its uniformity. • The Ca–P precipitation process depends on the pH of the medium, lower pH value maintains solubility.
		Oxygen	• Components of PNS are subject to oxidative degradation in the presence of oxygen. • PUFAs could be oxidized to harmful lipid hydroperoxides.
		Light	• Light protection measures protect light sensitive compounds, such as photosensitive vitamins, from photo-oxidation. • Exposure to daylight is more hazardous than artificial light due to the UV wave spectrum involved.
		Temperature	• Standard refrigeration temperatures increase the storage life of PNS. • Lipid peroxidation, and Ca–P precipitation may be accelerated by the increase of ambient temperature before and during the use of PNS.
		Preparation and mixing order	• The mixing order of the PNS compounds is important. • Adding lipids first to the glucose compartment may increase lipid degradation due to high acidity of the dextrose solution before additional buffering compounds are added.
		Coinfusion/addition of drug	• The addition, or concurrent administration, of the IVM to PNS increases the risks of the stability and incompatibility problems.
		Infusion sets, and containers	• Infusion sets and containers also play a role in the stability of PNS in terms of light protection, oxygen barriers, and direct interactions.

Abbreviations: AAs, amino acids; Ca–P, calcium–phosphate; IVM, intravenous medication; LEs, lipid emulsions; PNS, parenteral nutrition solutions.

#### Complexity of formulations

The intrinsic complexity of PN formulations represents a primary source of risk. PN admixtures are usually composed of components manufactured by different pharmaceutical companies, and even products with the same generic name may vary in formulation composition and physicochemical properties. Among all components, lipid emulsions—thermodynamically unstable colloidal systems—are the least stable constituents in advanced PN formulations. Their physical stability depends on the balance between electrostatic repulsion and van der Waals attraction at the droplet surface [217]. Once this balance is disrupted, processes such as flocculation, creaming, coalescence, and phase separation may occur [23].

Zhao et al. [218] compared the physical stability of 5 commercially available MCT/LCT-based lipid emulsions in total nutrient admixtures (TNAs) containing high concentrations of electrolytes and found significant differences within 72 h. Similarly, Rogulska et al. [219] demonstrated that 2 AA solutions exerted different effects on lipid peroxidation concentrations in 3 types of lipid emulsions within TNAs. Furthermore, Ross et al. [220] reported that most pediatric drug–lipid emulsion combinations were Y-site compatible, except 2 mg/mL cisatracurium with Intralipid and 2 mg/mL gentamicin with SMOFlipid. Even when individual formulations meet pharmacopeial standards, differences among brands or component combinations can still cause instability or incompatibility. Therefore, both internal formulation factors and external interactions must be carefully considered during PN preparation and administration.

#### Heterogeneity of research methodologies

The heterogeneity of research methodologies remains a major limitation to the clinical applicability of current findings. Most existing studies have focused on assessing physical stability, in which the MDD and PFAT5 are 2 critical parameters reflecting lipid emulsion stability and intravenous safety. Among them, PFAT5 has been recognized as a more sensitive and reliable indicator of emulsion stability [221,222]. However, most published studies have reported only MDD, neglecting PFAT5, which may underestimate potential risks. Substantial variations in analytical instruments, detection methods, formulation compositions, and experimental conditions further compromise the comparability and generalizability of results. Otero-Millán et al. [217] emphasized that methodological heterogeneity in assessing lipid emulsion stability directly undermines data reliability. Unger and Holzgrabe [223] recommended hydrophilic interaction chromatography coupled with mass spectrometry as a preferred method for analyzing AA stability.

Table 7 summarizes the major methodologies used for evaluating PN stability and compatibility. Overall, the current research landscape remains fragmented, with inconsistent parameter selection and limited clinical relevance. Some domestic studies have not strictly followed pharmacopeial standards, showing outdated analytical approaches and insufficient instrument adaptability. More importantly, no validated models have yet established the correlation between physicochemical parameters and clinical outcomes, leaving a critical evidence gap that limits the translation of research findings into clinical decision-making. Such a fragmented and nonstandardized methodological framework fails to meet clinical practice needs. There is an urgent need to establish unified, systematic, and cross-platform multidimensional evaluation standards and to strengthen the linkage between basic research and clinical outcomes to enhance both research quality and practical applicability.

**TABLE 7:** Current methodologies for evaluating the stability and compatibility of parenteral nutrition formulations.

Study object	Study item	Main instruments or methods
PNS	Appearance [224]	Visual inspection, Tyndall effect
	Microscopy [225]	Scanning electron microscopy
	pH	pH meter
	Osmolality	Freezing point osmometer
	Insoluble particles [226]	Light obscuration, microscopic counting
	Microbiological analysis [227]	Sterility testing
	Turbidity [228]	Turbidimeter
	Density [14,229]	Densitometer
	Viscosity [14,229]	Viscometer
Lipid emulsion [217,218]	Zeta potential	Laser Doppler electrophoresis
	Particle size and distribution (MDD, PFAT5, PDI)	Microscopic counting, Coulter counter, dynamic light scattering, laser diffraction, light obscuration, photon correlation spectroscopy, single particle optical sensing
	Content determination [219,230,231]	UV–Vis spectrophotometry, HPLC, GC–MS
	Peroxides [232,233]	Potentiometric titration, thiobarbituric acid assay, lipid peroxidation kits
	Free fatty acids [211]	Spectrophotometry
Amino acids [223]	Content determination	RP-HPLC, amino acid analyzer
Glucose	Content determination	Spectrophotometry
Vitamins [234]	Content determination	Iodometric titration, spectrophotometry, NMR, HPLC, HPLC-MS
Trace elements	Content determination	Atomic absorption spectroscopy, ICP-MS
Electrolytes	Content determination	Spectrophotometry
Packaging materials, Y-site administration, nonnutritive drugs	Included above items	Same as above

Abbreviations: GC-MS, gas chromatography-mass spectrometry; HPLC, high-performance liquid chromatography; HPLC-MS, high-performance liquid chromatography-mass spectrometry; ICP-MS, inductively coupled plasma mass spectrometry; MDD, mean droplet diameter; NMR, nuclear magnetic resonance; PDI, polydispersity index; PFAT5, percentage of fat residing in globules >5 μm; PNS, parenteral nutrition solution; RP-HPLC, reverse phase HPLC; UV-Vis, ultraviolet-visible.

### Special populations

Given the marked heterogeneity in metabolic demands, organ function, and risk profiles across different special populations, appropriate dosing strategies and monitoring parameters are essential to ensure the safety and effectiveness of PN therapy. Table 8 summarizes recommended macronutrient dosing ranges and key monitoring considerations for neonates, critically ill adults, and patients receiving HPN. In addition, micronutrient dosing recommendations for neonates and adults are detailed in Table 9.

**TABLE 8:** Macronutrient dosing and monitoring in special populations.

Population	Dosing	Monitoring^1^
Neonates [26,235,236]		
Term infants	Glucose: 2–5 → 5–10 mg/kg/min (max 12 mg/kg/min) AA: 1.5–2.5 → +1.0–1.5 g/kg/d (max 2.5–3.0 g/kg/d) Lipids: 1.0–2.0 → +0.5–1.0 g/kg/d (max 3.0–3.5 g/kg/d)	Body weight, length, head circumference; growth curve
Preterm infants	Glucose: 4–8 → 8–10 mg/kg/min (max 12 mg/kg/min) AA: as term infants (max 3.0–3.5 g/kg/d) Lipids: as term infants	
Adult critically ill patients		
General critically ill patients [24,237]	Glucose: ≤5 mg/kg/min AA: 1.2–2.0 g/kg/d Lipids: ≤1.5 g/kg/d	P, Mg; catheter site
Acute/subacute liver failure [238,239]	Glucose: target 8.3–10.0 mmol/L AA: 1.2–1.5 g/kg/d (hepatic encephalopathy) As above	Blood glucose every 2 h; NH_3_, coagulation, bilirubin
AKI/CKD (without RRT) [101]	As above AA: 1.0–1.3 g/kg/d	K^+^, P, Mg^2+^, acid-base; strict I/O
On RRT [101]	As above AA: IRRT 1.3–1.5 g/kg/d, CRRT 1.5–1.7 g/kg/d (≤2.5 g/kg/d if needed)	K^+^, P, plasma AA; adjust protein/electrolytes per RRT
HPN patients[240,241]	Glucose: ≤5–7 mg/kg/min AA: 0.8–1.4 g/kg Lipids: ≤1 g TG/d (if HPN >6 mo), ensure EFA supply	Every 3–6 mo: body composition, hydration, energy/fluid balance Annually: vitamins, trace elements, bone metabolism, BMD

Abbreviations: AA, amino acids; AKI, acute kidney injury; BMD, bone mineral density; CKD, chronic kidney disease; CRRT, continuous renal replacement therapy; EFA, essential fatty acids; HPN, home parenteral nutrition; I/O, intake/output; IRRT, intermittent renal replacement therapy; RRT, renal replacement therapy; TG, triglycerides.

1 General monitoring for all PN patients: blood glucose, lipid profile, liver and kidney function, electrolytes, and catheter insertion site (infection, swelling, leakage) should be regularly assessed unless otherwise specified.

**TABLE 9:** Micronutrient dosing in neonates and adults (per day).

Micronutrients	Neonates [184,242]		Adults [4]
	Term infants	Preterm infants	
Fe	50–100 μg/kg	200–250 μg/kg	≥1 mg
Zn	250 μg/kg	400–500 μg/kg	3–5 mg (without abnormal losses), major burns >20% BSA 30–35 mg
Cu	20 μg/kg	40 μg/kg	0.3–0.5 mg, severe deficiency 4–8 mg
Se	2–3 μg/kg	7 μg/kg	60–100 μg
Cr	0 μg/kg	0 μg/kg	≥10 μg, insulin-resistant critically ill patients 3–20 g/h
Mo	0.25 μg/kg	1 μg/kg	19–25 μg
Mn	≤1 μg/kg	≤1 μg/kg	55 μg
I	1 μg/kg	1–10 μg/kg	130 μg
Vitamin A	500–1000 IU/kg	700–1500 IU/kg	800–1100 μg RE
Vitamin D	40–150 IU/kg	80–400 IU/kg	≥200 IU (5 μg)
Vitamin E	2.8–3.5 IU/kg	2.8–3.5 IU/kg	≥9 mg
Vitamin K	10 μg/kg	10 μg/kg	150 μg Vitamin K-1
Vitamin B-1	350–500 μg/kg	350–500 μg/kg	≥2.5 mg, emergency or intensive care patients 100–300 mg
Vitamin B-2	150–200 μg/kg	150–200 μg/kg	3.6–5.0 mg
Niacin	4.0–6.8 mg/kg	4.0–6.8 mg/kg	≥40 mg
Vitamin B-6	150–200 μg/kg	150–200 μg/kg	4–6 mg
Folate	56 μg/kg	56 μg/kg	400–600 μg
Vitamin B-12	0.3 μg/kg	0.3 μg/kg	≥5 μg
Vitamin B-5	1–2 mg/kg	2.5 mg/kg	≥15 mg
Biotin	5–8 μg/kg	5–8 μg/kg	60 μg, additional amounts are available for RRT
Vitamin C	15–25 mg/kg	15–25 mg/kg	100–200 mg, critical illness during acute phase of inflammation 2–3 g

Abbreviations: BSA, body surface area; RE, retinol equivalent; RRT, renal replacement therapy.

#### Neonates and pediatric patients

Neonates and pediatric patients have immature organ development, and their nutritional requirements vary with age, growth status, and clinical condition. Compared with adults, they typically require PN formulations of smaller volume but higher nutrient concentrations [243,244]. Due to their reduced hepatic enzyme activity and low glomerular filtration rate, medication errors in this population may lead to either toxic accumulation or therapeutic failure [245]. In clinical practice, the limited availability of pediatric-specific PN products often necessitates the dilution of adult formulations, which increases the risks of dosing errors, resource waste, and microbial contamination [246]. An observational study by Akour et al. [247] reported that neonates receiving PN frequently experienced drug-related problems, primarily due to inadequate PN monitoring and dosing errors. Lipid emulsions are the most common PN components associated with medication errors [248]. Current lipid emulsions fail to fully meet the unique fatty acid requirements of preterm infants, and both soybean oil–based and FO–based emulsions carry potential safety risks [40,249].

Pediatric patients also face heightened risks regarding PN stability and compatibility, largely due to limited venous access and the frequent need for Y-site coadministration with other intravenous medications [246]. Several studies have shown that pediatric PN formulations can maintain acceptable physicochemical stability under recommended storage conditions [210,229]. However, a separate review summarized Y-site compatibility studies between intravenous drugs and PN solutions in pediatric populations, finding that among 55 tested drugs, 56% were compatible, 13% incompatible, and 31% yielded inconclusive results [250]. These findings suggest that although certain PN formulations exhibit acceptable storage stability, the uncertainty associated with coadministration remains significant.

Moreover, pediatric patients require higher calcium and phosphate intake to support bone development; however, the limited solubility of calcium and phosphate often prevents PN formulations from meeting these requirements [244]. Prescription review studies have identified electrolyte dosing and calcium–phosphate incompatibility as common sources of intervention in neonatal PN prescriptions [251]. Experimental studies further suggest that the choice of AA formulation and the inclusion of cysteine can enhance calcium–phosphate solubility in pediatric PN admixtures [252,253]. Collectively, these data highlight the importance of formulation selection and compatibility assessment to improve the stability and safety of pediatric PN.

#### Patients receiving HPN

HPN serves as a life-saving therapy for patients with chronic intestinal failure, and its prevalence continues to increase worldwide. However, the complexity of its use and management poses major challenges for healthcare professionals, patients, and caregivers [254]. HPN involves the transition of PN administration from the hospital to the home setting and typically requires long-term maintenance, which markedly increases the risk of PN-associated metabolic complications, such as hyperglycemia, electrolyte imbalances, and IFALD [255]. HPN is primarily indicated for patients with inflammatory bowel disease, short bowel syndrome, intestinal obstruction, radiation enteritis, mesenteric ischemia, and postoperative complications. Formulating an HPN prescription requires comprehensive consideration of the patient’s metabolic demands, nutritional status, organ function, and underlying disease [240]. However, there is currently little evidence to guide the formulation of macronutrients and micronutrients for HPN patients [13]. A single standardized HPN admixture rarely meets individualized needs; therefore, a thorough assessment and personalized regimen are essential for each patient.

Controversies surrounding lipid emulsions and MCBs remain prominent. HPN patients are particularly sensitive to lipid peroxidation. Some studies have suggested that SMOFlipid may be preferable for patients with comorbidities, whereas ClinOleic could be used in those without [256]. Although several randomized controlled trials have demonstrated that FO-containing mixed-oil emulsions (e.g., SMOFlipid) improve liver function parameters in patients with IFALD [42,257], a prospective cohort study reported that the use of mixed-oil emulsions might be associated with an increased hospitalization rate [258]. These findings highlight the need to consider both comorbid conditions and metabolic characteristics when selecting lipid emulsions for HPN therapy.

In addition, the standardization of HPN formulations varies across regions. Some centers use customized compounded PN admixtures, whereas others prefer commercially available MCBs [259,260]. Differences in formulation sources and preparation conditions between hospital and home environments—together with factors such as transport, storage, and ambient conditions—may further increase the risks of instability and incompatibility. MCBs have become increasingly popular due to their convenience and improved stability; however, their fixed composition limits the degree of nutrient individualization [261,262]. Moreover, currently available micronutrient preparations may not be suitable for all HPN patients, posing risks of deficiency or contamination [188,197,263,264]. Practical challenges such as maintaining aseptic technique in the home environment and inappropriate timing of micronutrient supplementation may also affect the stability of PN admixtures. Therefore, the development of comprehensive, multidisciplinary HPN management programs is urgently needed. Such programs should include standardized procedures, professional training, and patient education to enhance adherence, ensure safety, and optimize clinical outcomes [255,265].

### Manufacturing and regulation of PN formulations

The clinical application of PN formulations is constrained by the stability of supply chains, the maturity of manufacturing processes, and the integrity of regulatory systems. First, interruptions in raw material supply chains, insufficient manufacturing capacity, and factors such as natural disasters and the COVID-19 pandemic have led to widespread global shortages of PN components, including lipid emulsions, vitamins, and trace elements [9,23]. PN shortages are particularly prominent in the United States, potentially increasing the risk of medication errors and waste of medical resources. ASPEN has issued specific recommendations and precautions for various PN component shortages [246,[266], [267], [268]]. A global survey indicated that, even in countries reporting adequate PN supply, constraints in formulation options, AA diversity, and access to multiple-oil lipid emulsions and micronutrients remained [269]. To address micronutrient shortages, Joly et al. [270] proposed centralized compounding, symptom-based supplementation, enhanced monitoring, and individualized replacement strategies, and urged relevant professional associations to proactively report shortages. Molotsky et al. [271] attempted to substitute calcium gluconate with calcium chloride–sodium gluconate in response to the shortage, and found that administration via Y-site infusion may be a feasible option for infants receiving PN therapy.

Second, substantial differences exist among countries regarding legislation and regulatory policies for PN formulations, and many nations have yet to establish explicit regulations [16]. In the United States, the FDA classifies PN formulations as drugs; therefore, market authorization for new products requires comprehensive, controlled research data to demonstrate safety and efficacy [272]. However, this stringent approval process often delays the market entry of innovative products, limiting their potential to alleviate shortages. Notably, due to the shortage of trace element formulations, the FDA has categorized certain products as “unapproved marketed drugs,” meaning they lack FDA oversight for manufacturing, safety, and efficacy, as well as FDA-approved labeling information [272]. ASPEN has expressed serious concern over this issue. On reviewing existing parenteral vitamin and trace element products, ASPEN found that certain trace elements in some formulations may reach potentially toxic concentrations and emphasized the urgent need for the development and application of choline and carnitine as essential nutrients [3,180,183].

By contrast, Europe and several other regions offer a greater variety of PN formulations, and drug shortages are relatively uncommon. However, in some areas, the prevalence of generic products results in inconsistent manufacturing quality and standards, posing major challenges for regulatory authorities. Variations in excipients among generics may trigger pseudoallergic reactions and compromise bioequivalence compared with originator products, raising safety concerns [144,273,274]. In addition, in many countries, ready-to-use AIO PN formulations must comply with Good Manufacturing Practice standards and require regulatory authorization. However in some regions, the compounding of temporary PN admixtures is not subject to specific regulatory oversight [275]. Therefore, the global PN market faces a dual challenge: product shortages on one hand and substandard quality risks on the other.

Finally, the production of advanced PN formulations relies heavily on sophisticated manufacturing technologies and stringent quality control systems. Different types of PN formulations have distinct technical and equipment requirements, making it crucial to ensure consistency and safety across production batches. Batch-to-batch differences in antioxidant (e.g., α-tocopherol) content have been reported in commercial lipid emulsions [276], whereas deviations between prescribed and prepared PN admixtures have also been observed in clinical settings, particularly in nurse-compounded neonatal PN [277]. To address these challenges, Quality by Design (QbD) manufacturing strategies and optimized process controls have been proposed to enhance emulsion stability and safety [123,221].

In addition, improper mixing of PN solutions may generate insoluble particulates that can deposit in the lungs, liver, and reticuloendothelial system. Particles >5 μm can obstruct pulmonary capillaries, leading to pulmonary embolism [278]. Inline filters play a crucial role in reducing insoluble particles during PN administration. ASPEN recommends the use of 1.2-μm filters for TNA, glucose–AA mixtures, and lipid emulsions, although clinical adoption remains limited [279]. To address this issue, Cresi et al. [215] designed a randomized controlled trial to evaluate the safety impact of inline filters in neonatal central venous lines. If shown to significantly reduce adverse events, widespread use of inline filtration in neonatal ICUs could become a recommended standard.

## Future Perspectives on Advanced PN Formulations

Advanced PN formulations have made remarkable progress in formulation design, component selection, delivery system optimization, and industrial standardization. Significant advances have been achieved, particularly in lipid emulsions, combined vitamin and trace element preparations, and MCB systems, all of which demonstrate promising clinical potential. However, current PN formulations still face multiple challenges, including the complexity of available products, insufficient evidence-based selection criteria, risks related to stability and compatibility, inadequate adaptation for special populations, and limitations in supply chain and regulatory systems.

Future research should focus on establishing a multidimensional evaluation framework for stability and compatibility, enabling precise selection of core components and optimization of dosage forms. Efforts are also needed to develop specialized formulations tailored for specific populations while improving quality supervision and technical standards. Strengthening evidence-based studies on the safety and efficacy of PN formulations and enhancing translational links from fundamental mechanisms to clinical outcomes will be crucial. Only through multidisciplinary and cross-sector collaboration can the successful translation of advanced PN formulations from laboratory research to clinical practice be achieved, ensuring clinical efficacy and safety while meeting patients’ diverse and individualized nutritional needs.

With the advancement of precision medicine and nutritional science, the research and application of advanced PN formulations are expected to follow a positive trajectory. Future efforts should emphasize the precise combination design and individualized optimization of key PN components—lipid emulsions, AAs, vitamins, and trace elements—while developing formulations better suited to the metabolic characteristics of special populations, such as pediatric patients and those requiring HPN. Integration of metabolomics and big data analytics may further facilitate the development of personalized nutrition support strategies. For instance, the development of highly safe sulfur dioxide–free AA injections, novel carrier-based multivitamin systems, and standardized trace element combinations represents key directions for future innovation.

A comprehensive evaluation system covering physical, chemical, and microbiological stability should be established, integrating traditional quality parameters with environmental factors such as temperature, pH, and light exposure, to enable full-spectrum monitoring of PN component stability and compatibility. At the same time, unified and stringent quality control and regulatory systems should be developed, with strengthened safety assessments for generic formulations and enhanced international regulatory harmonization, to mitigate product shortages and quality variability. Moreover, advances in drug delivery technologies—such as lipid emulsions, mixed micelles, high-pressure homogenization, and automated compounding—should be leveraged to further optimize manufacturing processes and ensure batch-to-batch consistency and safety. Finally, more high-quality, multicenter, and long-term clinical trials are urgently needed to correlate physicochemical indicators with patient outcomes, thereby providing robust data support and evidence-based foundations for the clinical translation of advanced PN formulations.

## Author contributions

The authors’ responsibilities were as follows – Y. Zhu, XB, GX: conceived the study idea, designed the protocol, and critically revised the manuscript; MZ, XZ: drafted the initial manuscript; MZ: summarized the tables; Y. Zhou: prepared the figures; Y. Zhou, JZ, LZ, SZ: contributed to manuscript revision; Y. Zhu, XB, GX: provided overall guidance; and all authors: read and approved the final manuscript and agreed to be accountable for all aspects of the work.

## Data availability

Data described in the manuscript, codebook, and analytic code will be made available upon request pending application and approval.

## Funding

This work was supported in part by grants from the National Natural Science Foundation of China (Grant nos. 82102917); Jiangsu Provincial Double-Innovation Doctor Program (PF-302-2021); 2024-Shining Across China-Medicinal Research Fund (Z04J2023E095); The Nanjing Medical Science and Technology Development Program (nos. YKK23104); State Key Laboratory of Advanced Drug Delivery and Release Systems (Grant no. DSQZ-QN-20250106).

## Declaration of generative AI and AI-assisted technologies in the writing process

During the preparation of this work, the author(s) used ChatGPT (OpenAI) in order to improve language phrasing, grammar, and overall readability. After using this tool/service, the author(s) reviewed and edited the content as needed and take(s) full responsibility for the content of the publication.

## Conflict of interest

The authors report no conflicts of interest.
